# Recurrent Ureterosciatic Herniation Causing Right Obstructive Uropathy

**DOI:** 10.7759/cureus.57128

**Published:** 2024-03-28

**Authors:** Eduardo García-Rico, Luis Enrique Ortega-Polledo, Diego M Carrion, Ana Quiroga-Valcarcel, Laura Fernández Hernández

**Affiliations:** 1 Urology, Hospital Universitario Torrejon, Madrid, ESP; 2 Urology, Hospital Universitario Clínico San Carlos, Madrid, ESP; 3 General and Colorectal Surgery, Hospital Universitario Príncipe de Asturias, Madrid, ESP

**Keywords:** laparoscopic-assisted hernia repair, obstructive uropathy, pelvic floor disorder, ureteral hernia, sciatic hernia

## Abstract

Sciatic hernias are unusual, challenging to diagnose, and can present some treatment dilemmas. Sciatic hernias containing the ureter are even less common. Symptoms are variable from renal fossa pain, mild or severe pelvic pain, neuropathic pain, or dysesthesias. Although stenting alone can be a treatment option for this condition, in cases where symptoms or ureteral obstruction relapse after initial treatment, sciatic hernioplasty must be considered as the definitive treatment.

This article presents the case of a female patient who presented with a history of nonspecific abdominal pain and was diagnosed with a right-sided ureterosciatic hernia. This was managed with a ureteral stent for reduction of herniated ureteral content, but after recurrence, laparoscopic sciatic hernioplasty was performed. The patient was pain-free and without obstructive uropathy at the one-year follow-up.

## Introduction

Pelvic floor hernias are extremely rare [1]. Oftentimes, it is difficult to assess a correct diagnosis, which leads to therapeutic dilemmas. Among different types of pelvic floor hernias, sciatic hernias are by far the less common ones [2]. The sciatic hernia was first described by Papen in 1750. Most cases do not involve any organ. The presence of the ureter within the hernia sac is uncommon and even less frequently causes ipsilateral obstructive uropathy [3]. The first case involving the ureter was described by Lindbom in 1947 [4].

The sciatic foramen in the bottom of the pelvis is divided into the greater and lesser sciatic foramen by the sacrospinous and sacrotuberous ligaments. The greater sciatic foramen is divided again by the piriformis muscle into suprapyriform or infrapyriform hernias. Suprapyriform foramen contains the superior gluteal vessels and gluteal nerve. Infrapyriform foramen contains pudendal vessels and sciatic nerve. These hernias can produce symptoms related to the compression of the previously mentioned structures.

Sciatic hernias are commonly secondary to a partial loss of pelvic fascia, atrophy of the piriformis muscle, congenital defect, or a combination of both. Ureters usually herniate in the suprapiriformis compartment of the greater sciatic foramen [5].

## Case presentation

**Figure 1:**
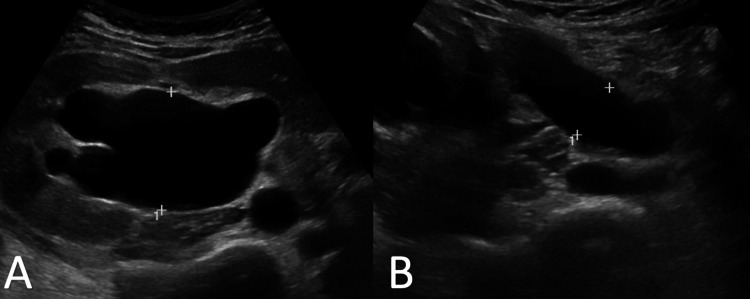
Initial abdominal ultrasound. A. Ultrasound image showing anteroposterior dilatation of renal pelvis of 4.4 cm. B. Ultrasound image showing ureteral dilatation of 1.87 cm.

A 62-year-old female, with no previous medical history of interest, presented to the emergency department with right flank pain and severe tenderness to palpation. The patient reported several months of intermittent pain in the right flank and ipsilateral hip of variable intensity, which in the previous weeks was localized more precisely in the right renal fossa. She also described paresthesia in the right lower limb and neuropathic pain that woke her from sleep in the previous days. First-line complementary tests included blood analysis (Table 1) and abdominal ultrasound (Figure 1), which showed complete right uretero-hydronephrosis without an underlying cause.

**Table 1:** Blood analysis results.

Blood analysis	Observed values	Reference values
Leukocytes	12,500 cells/mm^3^	3,900–10,200 cells/mm^3^
Neutrophils	83.2%	42–77%
Lymphocytes	11.5%	20–44%
Basophils	0.2%	0.0–2.3%
Eosinophils	0.7%	0.5–5.5%
Urea	62 mg/dL	15–50 mg/dL
Creatinine	0.72 mg/dL	0.5–1.1 mg/dL
Sodium	139 mEq/L	135–147 mEq/L
Potassium	4.9 mEq/L	3.5–5.1 mEq/L
C-reactive protein	0.42 mg/dL	0–0.5 mg/dL

**Figure 2:**
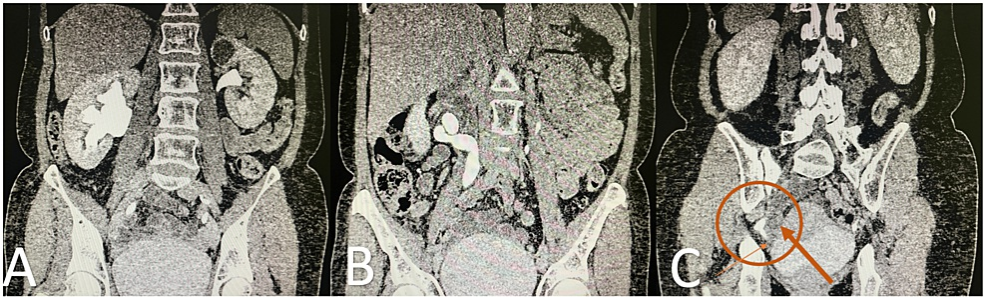
CT scan showing right severe hydronephrosis. A. Sagittal section showing hydronephrosis. B. Sagittal section showing ureteral dilatation. C. Marker: ureter herniating over the sciatic orifice.

A CT urography was requested to assess the cause of the uretero-hydronephrosis and revealed that the origin was a ureteral hernia in the sciatic space. The right kidney showed delayed contrast uptake and elimination with preserved renal parenchyma. There were no other possible intrinsic or extrinsic causes of ureteral obstruction (Figure 2).

**Figure 3:**
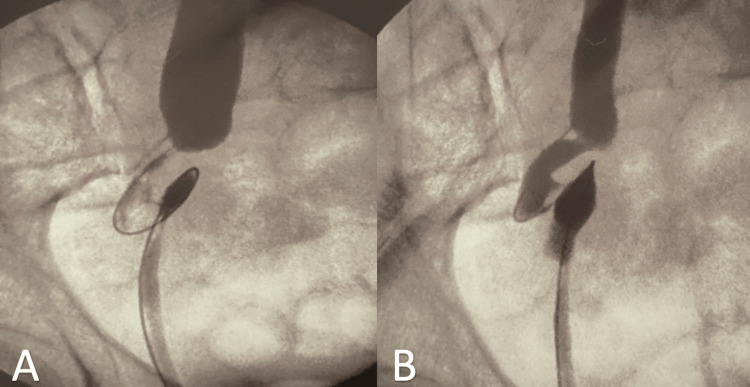
Intraoperative retrograde pyelography. Ureteral loop in the pelvic fossa.

With this diagnosis, a retrograde pyelography and exploratory ureteroscopy were performed under anesthesia in the operating room. An obstructive right megaureter with multiple loops without intraluminal lesions was observed, and retrograde drainage of the urinary tract was performed by a right double J catheter (Figure 3). The ureteral catheter was removed after one month. Three weeks after its removal, the patient was asymptomatic, and an abdominal ultrasound ruled out persistent hydronephrosis.

The patient came for consultation again referring to similar symptoms as the previous ones. An in-office abdominal ultrasound again showed right severe hydronephrosis. A new CT urography showed a recurrence of the right sciatic hernia, and the patient underwent a new retrograde pyelography and insertion of a double J stent.

**Figure 4:**
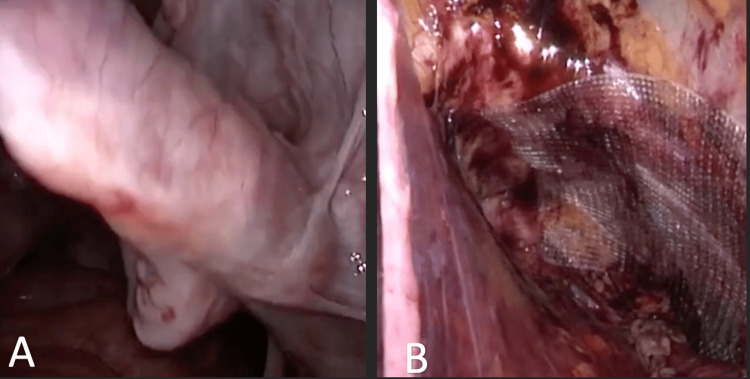
Transabdominal preperitoneal approach repair. A. Sciatic hernia sac. B. Final result after mesh placement.

The case was discussed in the multidisciplinary committee involving general surgery, and it was decided to perform a right sciatic hernioplasty through a transabdominal preperitoneal approach (Figure 4).

**Figure 5:**
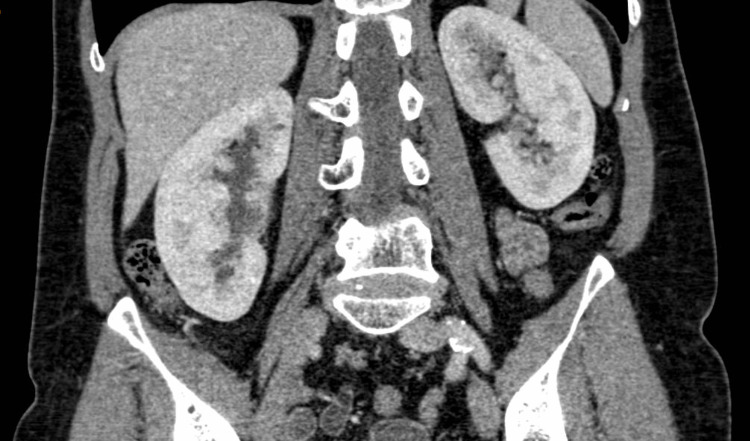
Two months follow-up CT scan. Sagittal section showing absence of pyelocaliceal dilatation.

A double J stent was removed one month after hernioplasty, and a control CT urography two months after the intervention showed a resolution of the uretero-hydronephrosis, symmetrical contrast uptake, and excretion (Figure 5).

At the one-year follow-up, the patient reported a resolution of all initial clinical symptoms, without lumbar pain and dysesthesias. The patient continues to experience mild discomfort occasionally in the right iliac fossa which does not prevent her from living a normal life.

## Discussion

The variable clinical presentation, the low prevalence, and the absence of extensive published literature on this topic (there are fewer than 40 cases of sciatic hernia involving the ureter published) make ureterosciatic hernia difficult to suspect and diagnose.

Diagnosis depends primarily on imaging tests. CT urography is the gold standard. The Curlicue sign is pathognomonic, and it is described as ureteric obstruction with U-shaped tortuosity through the sciatic foramen [6]. Early diagnosis is key to avoiding renal damage associated with chronic obstructive uropathy.

Based on the few cases published at present, the management of ureterosciatic hernias can be conservative at first via observation [7], waiting for spontaneous reduction of the hernia. This management has a low success rate and requires close follow-up to avoid renal damage. Second, many authors recommend ureteral catheterization as a measure of urinary obstruction release and reduction of the herniated ureteral contents. Once this is achieved, stent removal and re-evaluation can be attempted, although few cases have been described, and in some isolated cases, this has been a definitive treatment [8-10]. Ureteral catheterization can present technical difficulties due to the presence of loops in the hernial sac and severe ureteral dilation, which is why retrograde pyelography should always precede catheterization.

Finally, definitive hernia repair treatment has been described, especially in cases in which neuropathic pain appears due to compression of the nerve and vascular structures. This pain usually remains after urinary diversion and ureter reduction [11]. Several approaches have been described in the literature. The gluteal approach is performed through the gluteus maximus muscle. There is a higher risk of neurovascular injury and less exposure of the hernial sac. The laparoscopic or open transperitoneal approach provides better access to the hernial orifice and better management of included structures (ureter). The largest series of laparoscopic repairs of sciatic hernias included 19 patients and described the transperitoneal and mesh repair method as a safe and effective technique [12]. Therefore, despite the lack of data, it could be considered as the definitive treatment.

## Conclusions

Sciatic hernias are unusual, with a challenging diagnosis which can present some treatment dilemmas. Sciatic hernias containing the ureter are even less common. Symptoms are variable from renal fossa pain, mild or severe pelvic pain, neuropathic pain, or dysesthesias. Although stenting alone can be a treatment option for this condition, in cases in which symptoms or ureteral obstruction relapse after initial treatment, sciatic hernioplasty must be assessed as the definitive treatment.
